# Feasibility of protein-sparing modified fast by tube (ProMoFasT) in obesity treatment: a phase II pilot trial on clinical safety and efficacy (appetite control, body composition, muscular strength, metabolic pattern, pulmonary function test)

**DOI:** 10.1007/s12349-013-0126-2

**Published:** 2013-05-30

**Authors:** S. G. Sukkar, A. Signori, C. Borrini, G. Barisione, C. Ivaldi, C. Romeo, R. Gradaschi, N. Machello, E. Nanetti, A. L. Vaccaro

**Affiliations:** 1 Clinical Nutrition Unit, IRCCS San Martino University Hospital, National Cancer Institute, Largo R. Benzi 2, 16122 Genoa, Italy; 2 Biostatistical Unit, IRCCS San Martino University Hospital, National Cancer Institute, Largo R. Benzi 2, 16122 Genoa, Italy; 3 Respiratory Physiopathology Unit, IRCCS San Martino University Hospital, National Cancer Institute, Largo R. Benzi 2, 16122 Genoa, Italy; 4 DEXA Unit, Baluardo Institute, Genoa, Italy

**Keywords:** VLCD, PSMF, Tube feeding, Enteral nutrition, Protein, NEP, Ketogenic diet, Pulmonary function test, Hand grip strength, Obesity, Appetite, Enteral nutrition, Protein-sparing modified fasting by tube, ProMoFasT

## Abstract

Anecdotal data in the last few years suggest that protein-sparing modified diet (PSMF) delivered by naso-gastric tube enteral (with continuous feeding) could attain an significant weight loss and control of appetite oral feeding, but no phase II studies on safety and efficacy have been done up to now. To verify the safety and efficacy of a protein-sparing modified fast administered by naso-gastric tube (ProMoFasT) for 10 days followed by 20 days of a low-calorie diet, in patients with morbid obesity (appetite control, fat free mass maintenance, pulmonary function tests and metabolic pattern, side effects), 26 patients with a BMI ≥30 kg/m^2^ have been selected. The patients had to follow a protein-sparing fast by enteral nutrition (ProMoFasT) for 24 h/day, for 10 days followed by 20 days of low-calorie diet (LCD). The endpoint was represented by body weight, BMI, abdominal circumference, Haber’s appetite test, body composition by body impedance assessment (BIA), handgrip strength test, metabolic pattern, pulmonary function test. Safety was assessed by evaluation of complications and side effects of PSMF and/or enteral nutrition. In this report the results on safety and efficacy are described after 10 and 30 days of treatment. After the recruiting phase, a total of 22 patients out of 26 enrolled [14 (63.6 %) females] were evaluated in this study. Globally almost all clinical parameters changed significantly during first 10 days. Total body weight significantly decreased after 10 days (∆−6.1 ± 2; *p* < 0.001) and this decrease is maintained in the following 20 days of LCD (∆ = −5.88 ± 1.79; *p* < 0.001). Also the abdominal circumference significantly decreased after 10 days [median (range): −4.5 (−30 to 0); *p* < 0.001] maintained then in the following 20 days of LCD [median (range) = −7 (−23.5 to −2); *p* < 0.001]. All BIA parameters significantly changed after 10 and 30 days from baseline. All parameters except BF had a significant change after 10 days of treatment while the difference at 30 days was lower than at 10 days for TBW, FFM and MM with no significant differences from baseline for the last two characteristics. For VAS appetite the difference was significant after 10 days and the decrease in appetite was maintained at 30 days with no significant difference (*p* = 0.83) between 10 and 30 days. No significant differences in the first 30 days were detected for PA and for both left and right hand grip strength. Particularly, a significant reduction of 1.82 kg in FFM after 10 days was detected, but not after 30 days. In contrast, a decrease of 3.8 kg of BF is observed after 30 days. As far as the respiratory functional tests (RFT) are concerned, a significant difference at 10 days was globally observed for functional residual capacity (*p* = 0.012) and expiratory reserve volume (*p* = 0.025). There are no reported major complications and side effects resulting from the enteral nutrition or PSMF. In particular, cardiac arrhythmias have not been reported. From the clinical point of view the PSMF with naso-gastric tube (ProMoFasT) method appears safe, it is associated with a significant weight loss related to decrease of FM and not to loss of FFM and appetite decreases. It is relevant that the RFT are significantly improved after only 10 days suggesting the efficacy of this regime in short period, too. These preliminary data underline the necessity to increase the number of RCT for this method, which could represent a possible alternative to other methodologies, such as the intragastric balloon, in particular when it is recommended to improve RFT before bariatric, gynecological, orthopedic and lymphatic surgery.

## Introduction

The increase in obesity in western populations constitutes a significant health problem and in countries like the US, the prevalence is estimated to be about 18–20 %. In Italy, overall prevalence of obesity is approximately 9 % while the severe form affects 1 % of the population with a higher incidence in females. Obesity (especially if moderate–severe) creates significant morbility and mortality.

Currently available therapeutic strategies for the treatment of obesity are dietetic-behavioral, pharmaceutical and surgical. 

The last option is usually adopted in patients with severe obesity (>40 kg/m^2^) or with a BMI >35 kg/m^2^ and multiple related diseases, who are non-responders to the other available options.

Also if in the recent years new methods have been developed to obtain fast results before bariatric surgery, there exists clinical situations in which it could be advocated to reduce weight fastly [i.e., obese patients (BMI 30–45 kg/m^2^)], affected by comorbidities such as orthopedic diseases (coxarthrosis, gonarthrosis), moderate or severe respiratory (OSAS) diseases, patients that need to loose weight quickly due to scheduled vascular or orthopedic surgery and are not eligible for bariatric surgery for esthetic or pneumological risk.

Even though intragastric balloon (IGB) is a possible medical choice since it is associated with an improvement/cure of the severe obesity, according to a recent cochrane, it is absolutely contraindicated in case of voluminous hiatal hernia, abnormalities of the pharynx and oesophagus, oesophagus varicose veins, use of anti-inflammatory or anti-coagulant drugs, pregnancy and psychiatric disorders [1]. Relative contraindications are oesophagitis, ulceration and acute lesions of the gastric mucous membrane [1]. Comparing IGB with conventional management, there is not convincing evidence of a greater weight loss [1]. Moreover, the relative risk for minor complications, for example gastric ulcers and erosions is significantly higher (÷0.06 %) but mortality rate is low (÷0.09 %) [1].

In the last few years some anecdotical data suggested that protein-sparing modified diet (PSMF) delivered by naso-gastric tube enteral (with continuous feeding) (ProMoFasT) for 15–21 days could obtain an important weight loss and control of appetite vs delivering by mouth but no controlled studies have demonstrated that this treatment could be safer or more efficient than protein sparing by mouth.

In fact, there are evidences suggesting the efficacy of protein-sparing modified fast (PSMF) in provoking a significant weight loss associated with a high loss of body fat [2]. This formula was developed in 1970 by a work group headed by Bistrian et al. [3] and it consists in the administration of high biological value protein (1.2–1.4 g/kg per day), carbohydrates = 40 g and vitamins and mineral supplement.

Recently, a very interesting study by Marseet-Baglieri et al. on rats have clearly shown that in the total absence of carbohydrates, the reduced insulin response tips the balance in favor of oxidation of lipids rather than depositing them by means of the inhibition of triglyceride synthesis and activation of triglyceride lipase by glucagon. Confirmation is provided by the fact that an increased calorie intake and adiposity is greater in rats given a free diet with 30 % of lipids and 56 % of carbohydrates compared to those undergoing a free diet with 50 or 70 % lipids without carbohydrates [4].

Moreover, it has been hypothesized that tube feeding could favor appetite control because there is evidence in support of the anorexigenic effect induced by naso-gastric tube feeding [5].

 Even if PSMF, under proper medical supervision, can achieve excellent results, this diet has never been studied, without carbohydrate intake and without tube feeding.

Therefore, the present phase II trial has been designed to verify the safety and efficacy of a protein-sparing modified fast (PSMF) administered by naso-gastric tube with protein intake 0.8–1 g/kg per day without carbohydrate intake (lower than 1 %) and an adequate intake of vitamins, electrolytes and fiber for 10 days, in patients with morbid obesity.

## Patients and methods

A total of 26 patients were enrolled for the study. To be included a BMI ≥30 kg/m^2^ was required. The patients were selected among those who had not previously responded to medical treatment and/or cognitive/behavioral therapy, who were candidates for invasive treatment such as the gastric balloon or surgery but refused these approaches.

Exclusion criteria were a severe grade active esophagitis, voluminous hiatal hernia (>5 cm), active phase gastric ulcer and/or duodenal ulcers, previous restrictive surgery of the gastrointestinal tract, Crohn’s disease, cancers, esophageal-gastro-duodenal hemorrhages in course or potential bleeding esophageal-gastric lesions, treatment with NSAIDs or anticoagulants, patients with psychiatric disorders or patients who are uncooperative, alcohol or drug-dependent patients, type I diabetes, type II diabetes in treatment with sulfonylurea, patients with liver, kidney or multi-organ failure.

Further, patients with eating disorders (the patients have been included only if they have undergone psychiatric assessment) were excluded, patients with contra-indication to enteral nutrition as chronic mechanical intestinal obstruction or semi-obstruction, acute mesenteric ischemia on a non-hypovolemic base, jejunal or intestinal fistulas (output 400 mL/day), severe changes to intestinal functionality secondary to enteropathy or insufficiency of the absorbent surfaces thus preventing the maintenance of an adequate nutritional state). 

The endpoints were: body weight; BMI; body composition, abdominal circumference; Haber’s appetite test; handgrip strength, biological tests and pulmonary function test.

### Handgrip strength (HGS)

HGS was measured by using the handy dynamometer (JAMAR^®^) by experienced personnel blinded to all clinical and biochemical data of the patients. The patients were instructed to produce as much handgrip pressure as possible by using the non-dominant hand. The measurements were repeated three times, and the highest score was recorded in kilograms.

### BIA

A single tetrapolar BIA measurement of resistance (*R*) and reactance (Xc) was taken at a fixed frequency of 50 kHz between the right wrist and ankle (standard placement of surface electrodes) with a body impedance analyzer (BIA 2000-S; Akern srl, Firenze) while the subjects were in a supine position on a nonconductive surface. The determination of body composition parameters were assessed according to the literature [6].

### Lung function measurements

Inspired and expired volumes were measured by using a mass flowmeter (CareFusion; VIASYS-SensorMedics Inc., Yorba Linda, CA, USA) with numerical integration of the flow signal. Vital capacity (VC) and forced expiratory volume in 1 s (FEV1) were measured according to the American Thoracic Society/European Respiratory Society (ATS/ERS) recommendations [7]. Functional residual capacity (FRC) was measured by whole-body plethysmography V62J; CareFusion, VIASYS-SensorMedics Inc.) during panting against a closed shutter at a frequency ranging from 0.5 to slightly <1.0 Hz. Residual volume (RV) was calculated by subtracting a linked expiratory reserve volume (ERV) from FRC and total lung capacity (TLC) by adding the subsequent inspiratory VC [8]. Predicted values for spirometry and lung volumes were from Quanjer et al. [9].

Lung diffusing capacity for carbon monoxide (DLCO) (Vmax22D; CareFusion, VIASYS-SensorMedics Inc.) was measured according to the ATS/ERS task force [10] and predicted values were from Cotes et al. [11].

### Compliance and safety

The patients described complications and side effects of tube feeding treatment in a diary. During the 6-h admissions in Day Hospital and then at home (following the usual protocol for Home enteral Nutrition), the patients were monitored for safety (adverse events, complications physical exams). During the first 10 days patients were monitored for the following side effects: enteral nutrition ones (abdominal distension and pain, diarrhea, vomiting, reflux, naso-gastric tube obstruction, de-positioning or accidental removal of the naso-gastric tube, others) and PSMF ones (cardiac arrhythmia, ketosis, hypoglycemia, others). Patients were assessed during initial observation and on a monthly basis.

### Materials

The necessary materials has been sponsored by Nestlé Health Science which supplied the devices and clinical nutrition formula and protein oral feeding powder for the artificial nutrition (Pumps; Infusion kits; Naso-gastric tubes; Tube medication kits); Resource^®^ Instant Protein, 1 g/kg/ideal body weight with BMI = 25/day × 10 days; modified guar gum fiber (Optifibra^®^), 20 g/day/pt × 10 days; potassium salt, 20 g/day × 10 days; multivitamin complete support (2/day × 10 days). The Insurance will be covered by the same company.

## Diet-food protocol during and after ProMoFasT

During the first 10 days the patients were fed with a PSMF treatment with the naso-gastric tube which was calculated according to the characteristics reported in Table 1.

**Table 1:** Nutrient requirements

Nutrients	Requirements/pro kg/day
Proteins (g)	1/kg ideal weight with BMI = 25
Fiber (g)	20
Sodium (mg)	32
Potassium (mg)	35
Calcium (mg)	4.4
Phosphorus (mg)	4.7
Iron (µg)	56
Zinc	46
Manganese	5.5
Vit A (retinol) (UI/µg)	3,300
Vit D_2_ (UI/µg)	200
Vit E (tocopherol) (UI/mg)	10
Vit C (ascorbic acid) (mg)	60
Vit B_1_ (thiamine) (mg)	1.4
Vit B_2_ (riboflavin) (mg)	1.6
Vit B_6_ (pyridoxine) (mg)	2
Vit B_12_ (cyanocobalamin) (µg)	2
Vit B_5_ (pantothenic acid) (mg)	6
Vit H (biotin) (µg)	0.15
Vit PP (niacin) (µg)	18
Folic acid (µg)	200
Vit K (µg)	70

The protein requirements were calculated by the following formula: protein = 1 g/kg ideal body weight (with an ideal body weight calculated on BMI = 25).

For the following 20 days the patients followed a low-calorie diet with a caloric deficit of about 10 % of their caloric needs calculated by means of Harris–benedict formula. The treatment and the diet has been given to patients and followed at home and were explained by a dietician. Moreover, some advice on changes in lifestyle was suggested.

### Naso-gastric tube placement and enteral nutrition at home

The naso-gastric tube (NGT) was placed according to the following procedure prescribed by the protocol. 

After an overnight fast, an 8 French polyurethane naso-gastric tube was placed according to best clinical practice. This procedure took place in day-hospital (Dietetics and Clinical Nutrition Unit of IRCCS San Martino University-Hospital, Genoa, Italy) from March 2011 to October 2011.

During the visit, the patient was supplied with the necessary material for enteral nutrition, a pump and enteral feeding controllers and they received a technical explanation regarding the use of the pump and any possible side effects (see informed consent).

Patients were managed at home after training as usual and routinely during ProMoFasT carried out at home by our hospital in accordance with the protocol.

Patients were supplied with a free-phone number for any technical problems related to the pump, the telephone number of the Hospital Unit during working hours (8 am–3 pm) and a telephone number for the head of the department for clinical problems arising outside working hours. 

After 10 days the tube were removed and enteral nutrition stopped.

### Ethics

The Ethics Committee for Medical Research in Genoa, Italy (n.reg CEA 125/10) approved the protocol in February 2011. The treatment followed was in accordance with the ethical standards of the Helsinki Declaration (1975, revised in 1983). Patients signed the informed consent before entering to the study.

### Statistical methods

Normality of distribution of measurements was assessed by mean of histograms, Skewness index and Shapiro–Wilk test for normality [12].

The global change of each clinical characteristic on first 30 days was separately investigated by linear mixed model (LMM) in which time was considered as fixed factor and the patient identifier as random effect. For spirometric characteristics paired samples Student’s *t* test was used to assess differences between baseline and 10 days.

A *p* of 0.05 was considered statistically significant. SPSS (IBM Corp, v.19) was used for computation.

## Results

After the recruiting phase a total of 22 patients out of 26 enrolled, with 14 females (63.6 %), were evaluated in this study. The basal characteristics of patients are reported in Table 2.

**Table 2:** Clinical characteristics at baseline

Clinical characteristics	Mean (SD)
Weight (kg)	120.67 (25.34)
Height (cm)	157.92 (35.83)
BMI (kg/m^2^)	43.98 (7.97)
Age	48.64 (10.10)
Abdominal circumference (cm)	127.08 (12.96)
VAS appetite	6.95 (1.42)
RZ (Ω)	396.14 (91.98)
XC (Ω)	49.23 (9.18)
PA (°)	7.21 (1.15)
TBW (kg)	52.70 (14.54)
TbW (%)	43.26 (5.88)
ICW (kg)	30.70 (9.70)
ICW (%)	58.17 (5.37)
FFM (kg)	70.89 (19.81)
FFM (%)	58.55 (8.08)
MM (kg)	50.60 (15.16)
MM (%)	41.86 (7.08)
ECM (kg)	29.07 (7.29)
BCM (kg)	42.12 (13.08)
BCM (%)	58.95 (4.58)
FM (kg)	49.81 (13.48)
FM (%)	41.52 (8.27)
Hand grip strength (right)	36.00 (13.96)
Hand grip strength (left)	32.24 (11.84)

*Rz* resistance, *Xc* reactance, *PA* phase angle, *TBW* total body water, *ICW* intracellular water, *FFM* fat free mass, *MM* muscle mass, *ECM* extracellular mass, *BCM* body cell mass, *FM* fat mass, *SD* standard deviation

Three patients (9 %) dropped out during the tube feeding period due to scarce compliance and 1 (3 %) for allergy to milk protein.

Total body weight significantly decreased after 10 days (∆ −6.1 ± 2; *p* < 0.001) and this decrease is maintained in the following 20 days of LCD (∆ = −5.88 ± 1.79; *p* < 0.001).

Also the abdominal circumference significantly decreased after 10 days [median (range) −4.5 (−30 to 0); *p* < 0.001] and this decrease is maintained in the following 20 days of LCD [median (range) = −7 (−23.5 to −2); *p* < 0.001].

All BIA parameters had a significant change in first 30 days from baseline (Table 3). All parameters except BF had a significant change after 10 days of treatment while the difference at 30 days was lower than at 10 days for TBW, FFM and MM with no significant differences from baseline for the last two characteristics. For VAS appetite the difference was significant after 10 days and the decrease in appetite was maintained at 30 days with no significant difference (*p* = 0.83) between 10 and 30 days. No significant differences in the first 30 days were detected for PA and for both left and right hand grip strength.

**Table 3:** Change of body composition at 10 and 30 days from baseline

Characteristics	10 days difference			30 days difference			Difference 10 days/30 days (*p* value)	Differences on overall change (*p* value)
	*N*	Mean (SD)/median (range)	*p* value	*N*	Mean (SD)/median (range)	*p* value		
BIA								
Weight (kg)	22	−6.1 (2)	<0.001	20	−5.88 (1.79)	<0.001	0.78*	<0.001
Abdominal circumference (cm)	19	−4.5 (−30 to 0)	<0.001	17	−7 (−23.5 to −2)	<0.001	0.006	<0.001
TBW (kg)	19	−4.3 (3.8)	<0.001	17	−1.84 (2.64)	0.012	0.016	<0.001
FFM (kg)	20	−5.9 (5.1)	<0.001	18	−1.82 (4.37)	0.058*	0.014	<0.001
BCM (kg)	20	−4.7 (5.4)	<0.001	18	−1 (6.48)	0.53*	0.056*	0.002
BF (kg)	20	−2 (−10 to 17.6)	0.10*	18	−4.8 (−16.4 to 5.8)	<0.001	0.002	0.002
VAS appetite	21	−2 (−7.5 to 0)	<0.001	20	−2 (−8 to 0)	0.001	0.83*	<0.001
Phase angle (°)	20	−0.4 (1.4)		18	0 (1.82)	–	–	0.88
Hand grip strength								
Right	17	−1 (−3 to 6)	–	16	−1.5 (−9 to 25)	–	–	0.20
Left	17	0 (−8 to 4)	–	16	0.78 (−6 to 27)	–	–	0.98

*SD* standard deviation

Particularly, a significant FFM reduction of 1.82 kg after 10 days was observed while not significant changes were detected after 30 days. In contrast, a decrease of 3.8 kg of BF is observed after 30 days.

Table 4 shows the mean values for characteristics of pulmonary function test at baseline and after 10 days of treatment.

**Table 4:** Standard lung function data obtained before and after 10 days of enteral protein tube feeding

Pulmonary function tests	Baseline	10 days	*p*
VC (L)	3.52 (0.91)	3.56 (0.95)	0.47
FEV_1_ (L)	2.66 (0.82)	2.69 (0.82)	0.67
FEV_1_/VC	0.73 (0.04)	0.72 (0.05)	0.93
TLC (L)	5.11 (0.71)	5.23 (0.83)	0.43
FRC (L)	2.12 (0.28)	2.32 (0.38)	0.013
RV (L)	1.53 (0.38)	1.67 (0.36)	0.73
ERV (L)	0.50 (0.32)	0.61 (0.33)	0.024
DL_CO_ (mL min^−1^ mmHg^−1^)	21.6 (5.13)	21.5 (6.24)	0.91
DL_CO_/V_A_ (mL min^−1^ mmHg^−1^ L^−1^)	4.76 (0.74)	4.81 (0.68)	0.49

Data are expressed as absolute numbers or means (SD) of  % predicted values

*VC* vital capacity, *FEV1* forced expiratory volume in 1 s, *TLC* total lung capacity, *FRC* functional residual capacity, *RV* residual volume, *ERV* expiratory reserve volume, *DLCO* single-breath diffusing capacity of the lung for carbon monoxide, *VA* alveolar volume

A statistically significant difference at 10 days was globally observed for FRC (*p* = 0.013) and ERV (*p* = 0.0254) with an increase of measured values.

 A decrease was also observed for values of BMI with a similar trend for time (*p* < 0.001) as found for weight. In Table 5 the biochemical data are shown.

**Table 5:** Biochemical modification at 30 days from baseline

Clinical characteristics	Baseline, mean (SD)	30 days, mean (SD)	*p* value
Glucose (mg/dL)	106.71 (29.26)	95.29 (24.18)	0.002
Insulin (mU/L)	21.96 (10.26)	11.37 (4.91)	<0.0011
Homa	5.58 (2.10)	2.74 (1.53)	<0.001
Creatinine (mg/dL)	0.86 (0.17)	0.86 (0.19)	0.74
GGT (U/L)	22.40 (12.26)	17.43 (5.77)	0.006
ALP (U/L)	190.57 (65.61)	66.43 (15.99)	<0.001
AST (U/L)	18.60 (6.69)	19.86 (3.13)	0.72
ALT (U/L)	24.20 (14.04)	21.14 (6.31)	0.28
Cholesterol (mg/dL)	256.50 (28.97)	243.3 (30.14)	0.033
Hdl (mg/dL)	50.67 (11.93)	54.00 (9.98)	0.07
Ldl (mg/dL)	127.83 (46.15)	104.00 (35.32)	0.056
Triglycerides	125.43 (60.93)	87.57 (44.09)	<0.001
Na (mEq/L)	141.67 (2.80)	140.57 (2.37)	0.28
K (mEq/L)	3.90 (0.36)	3.80 (0.50)	0.43
Cl (mEq/L)	104.00 (3.11)	101.86 (4.30)	0.10
Ca (mEq/L)	9.51 (0.34)	9.44 (1.02)	0.72
P (mEq/L)	3.01 (0.28)	3.10 (0.47)	0.99
Mg (mEq/L)	1.91 (0.22)	1.94 (0.21)	0.66

*SD* standard deviation

## Discussion

### Body composition and metabolic parameters

The actual experience demonstrates that PSMF with naso-gastric tube (ProMoFasT) is associated with weight reduction mostly due to fat reduction than fat free mass. 

In fact, after the first 10 days, TBW, FFM and BCM are strongly reduced while BF is not decreased, after 20 days of LCD the results are completely different and the BF is strongly decreased while TBW, FFM, BCM are less decreased. These results could depend on the variation of the hydration during the first 10 days and after the rehydration in the following 20 days.

Before weight loss, FFM hydration is usually high and overweight and obese individuals are usually overhydrated based on chemical cadaver analysis [13, 14]. 

After 10 days of ProMoFasT the patients are dehydrated and according to the literature BIA underestimated the changes in BF [15–17]. Finally, after 20 days of well-balanced low-calorie diet the hydration is quite restored and the measurements could be compared with the baseline.

Therefore, this could explain why the differences in delta BF and delta BF % are not observed after 10 days but only after 30 days.

In 2004 Buchholz and Schoeller posed serious questions regarding the substantial difference observed in terms of weight loss when comparing low-calorie, low-sugar and low-fat diets. Even though the authors concluded that from a physical point of view, a calorie is a calorie; they however highlighted that the factors adopted by Atwater in his experiments to calculate energy that can be metabolized by food were not exact, in so far as, this equation tends to over-estimate the amount of available energy and fails to take into account various factors which depend on the types of food, such as bio-availability, type of fiber, the level of intestinal absorption, etc. [18]. The authors further noted that the substitution of one macronutrient with another is not a perfectly identical phenomenon in terms of energy results [18].

This difference can be seen in high-protein low-carbohydrate diets which produce a superior weight loss of 2.5 kg after 12 weeks of treatment compared to high-protein diets alone. 

In fact the authors concluded that this axiom is not unquestionable and empiric reality demonstrates that low-carbohydrate diets produce a greater consumption of body fat, even though the reason for this is unclear [18].

In this regard a meta-analysis by Krieger et al. demonstrated how a low-carbohydrate high-protein diet (protein intake >1.05 g/kg/day–carbohydrate intake <35 % gain) has a positive effect on body mass and its composition irrespective of energy intake. From this meta-regression the authors concluded that the loss of body fat is optimal when carbohydrate intake is <41.4 % and protein intake equal to 1.20 g/kg/day [19]. On the other hand, the lost of fat free mass following a diet seems to be independent of the total calorie intake but only dependent on daily protein intake.

The data from the present study go further and clearly demonstrated that the hypothesis which a complete PSMF carbohydrate free could be associated with an important decrease of FFM is not true.

As a matter of fact there are literature reports on similar reduction of fat free mass after 4 weeks (as in the present experience) of diet different in nutrient composition, i.e., Roy et al. after a 2,023 kcal/daily hypoglycemic diet (43.1 % CHO) show a total weight decrease of 1.8 ± 0.5 kg, with a FFM decrease of 1.0 kg ± 2. and of FM % 0.4 ± 6.8; Rumpler et al. after a 1,544 kcal/day hypoglycemic diet (46 % CHO) a total weight decrease of −5.2 kg ± 1.2 with a FFM decrease of −1.3 ± 1.9 kg and of FM % −2.5 ± 1.3 FFT −3.9 kg ± 1.0 and Hoeger et al. after a 1,448 kcal/hypolipidemic diet (65 % CHO) a total weight decrease of −2.8 ± 3.4 kg with a FFM decrease of 0.7 kg ± 1.9 and of FM % −1.2 ± 1.1 [20–22].

Finally, a confirmation of the fat free mass maintenance is obtained by the observation that hand grip strength in unchanged after 10 days of ProMoFasT. HGS is an easy, quick, inexpensive and readily available bedside test. Previous study reported that HGS may be an indirect marker of body lean muscle mass and represents a surrogate method to evaluate it [23].

The data obtained by the present experience are the clinical demonstration of what obtained in the rat when carbohydrates are absent [4]. According to Marsset-Baglieri [4] experience, it is also possible that the complete absence of carbohydrate may have reduced insulin secretion and thus favored the utilization rather than the storage of ingested lipids.

 In other words, low-carbohydrate rather than high-protein diets may be the main factor responsible for reducing adiposity when the protein content of the diet is increased. Moreover, the lowered insulin secretion resulting from the absence of carbohydrate in diet can determine less postprandial fat deposition [24].

In the present experience, after 1 month, insulinemia significantly reduced and this is in agreement with a reduced fat synthesis. In this situation of severe energy restriction, subjects are constantly in a state of negative energy balance, i.e., no net lipid storage occurs [4]. 

When analyzing the metabolism during fasting it is important to understand which energetic substrates can be used in a low-carbohydrate ketogenic diet.

When exogenous glucose is scarcely available and hepatic glycogen reserves have been depleted, the body’s physiological response is to utilize amino acids and fats in order to produce energy and glucose. 

In order to meet energetic requirements during fasting, ketone bodies derived from the oxidation of fatty acids are mainly used. Among the tissues that can utilize this source of energy are: the heart, skeletal muscle, the kidneys and mammary glands during breast feeding.

Ketone bodies are used inside the mitochondria; therefore, cells that do not have them, i.e., erythrocytes and the retina cannot draw energy from them. Nevertheless, even the brain, which uses glucose as its main fuel, can adapt to using ketone bodies after more than 20 days of fasting. In fact, ketone bodies different from fatty acids can, given their hydrosolubility, overcome the haemato-encephalic barrier. Furthermore, apart from extreme and prolonged conditions, the central nervous system exclusively utilizes glucose, on average 120 g per day, which it must extract from circulating blood as it is impossible to accumulate a reserve. 

Moreover, during prolonged fasting or a low-carbohydrate ketogenic diet (LCKD), compensatory gluconeogenetic mechanisms are set in motion.

The most well-known response to this requirement is gluconeogenesis from amino acids which permits in the initial phase of fasting to maintain baseline glucose thanks to the breakdown of lean muscular mass.

It is proven that the contribution of gluconeogenesis to the production of glucose goes from 47 ± 4 % after 14 h, 67 ± 4 % after 22 h, and 93 ± 2 % after 42 h of fasting [25]. 

This process is antagonized in the presence of food-derived protein, in PSMF, which are partly used for gluconeogenesis and partly for endogenous protein synthesis.

Moreover, in parallel to amino acids and lactate another important substrate exists for glucogenesis which is often forgotten, that is glycerol coming from triglycerides. During prolonged fasting, 10 % of gluconeogenesis is represented by lipolysis of triglycerides with a very low efficiency equal to 5 %; consequently, from 80 g of triglycerides 8 g of glycerol and 4 g of glucose are formed.

For this reason, by not introducing carbohydrates into a diet 1,000 g of fat allow for the production of 50 g of glucose (the minimum quantity sufficient in preventing lipolysis and ketogenesis) which corresponds to a caloric intake of 9,000 kcal [26]. The contribution of glycerol to glucose synthesis as been estimated at around 3 % after nocturnal fasting [27] and 10 % after 60 h of fasting [28]. We can reasonably estimate that with a TEE equal to 2,000 kcal, 10 % of energy expenditure is derived from glucose originating from glycerol that is approximately 50 g which in turn are derived from the breakdown of 1,000 triglycerides/day.

The breakdown of triglycerides in order to supply glycerol is an extremely costly process from a thermodynamic point of view and could in part explain the difference in weight loss achieved in patients following a rigorous low-carbohydrate diet compared to those following a low-protein one. 

Furthermore, this phenomenon could be predominant in the obese population.

As previously reported, the obese tend to lose less lean mass compared to slim subjects when fasting, perhaps because of an inferior mitochondrial efficiency which in turn imposes a greater quantity of ketone bodies in circulation consequent to a superior concentration of circulating fatty acids [29].

From several studies including the Elia’s [30], we note that the effects of fasting vary among lean and obese subjects: first of all, the concentration of ketone bodies, during the first 3 days of fasting are doubled in lean subjects compared to obese ones. The ratio between 3-hydroxybutyrate and acetoacetate which is an index of the status of mitochondrial redox increases more rapidly in the lean compared to the obese; and finally after 60 h of fasting, oxidation of leucine increases more significantly in lean subjects.

To further confirm the previous data, Benedict’s historical data and more recent data from Elia and Forbes’ group, during a restrictive diet obese subjects lose, a proportionally inferior quantity of lean mass compared to lean subjects [31]. 

Therefore, if individual genetic characters determine the maintenance of fat free mass, it is also true that the relationship between macronutrients influences this process [32].

As far as the metabolic parameters are concerned, after 4 weeks fasting glucose [106.71 ± 29.26 vs 95.29 ± 24.18 mg/dL (*p* = 0.001534)]; and insulin (from 21.96 ± 10.26 to 11.37 ± 4.91 mU/L) (*p* = 0.000726) are significant statistically reduced while hyperinsulinism is reverted (HOMA: 5.58 ± 2.10 vs 2.74 ± 1.53; *p* = 0.000165). 

A light degree of biochemical cholestasis demonstrated by high elevated values of ALP probably due to metabolic syndrome steatosis, is reduced after the treatment [190.57 ± 65.61 vs 66.43 ± 15.99 U/L (*p* = 0.000002)].

 Hyperlipemia, frequently associated with obesity, is observed and there is a reduction of total cholesterol 256.50 ± 28.97 vs 243.3 ± 30.14 mg/dL (*p* = 0.033242) and triglycerides 125.43 ± 60.93 vs 87.57 ± 44.09 mg/dL (*p* = 0.000012) following ProMoFasT.

These data are, in the short term, in agreement with previous experiences with PSMF with an elemental diet (protein 78 %, carbohydrates 19.6, fats 2.6 %) even if not completely a glucidic in a short preoperative treatment with a similar formula assumed by mouth [33].

### Appetite

With regard to appetite, our data confirm the anorexigenic effect of the ketogenic diet, but it is possible that the naso-gastric tube feeding could play a role, too.

 It is clear, however, from the available literature that a loss of appetite can be observed when the concentration of ketones is >4 mmol/L [34, 35] and this was observed in all the treated patients. But, according to Rolls and Roe [5] it has been demonstrated, under experimental conditions, that subjects with a naso-gastric tube ingest less food and reduce the contribution of food compared to those without a tube even if the infusion is simulated (that is, in patients who have a tube to which the infusion mixture is simulated) compared to those in the control group who are without a tube [5].

According to the present experience, appetite was completely suppressed during tube feeding since the first 2 days which is different from the literature data which show that it needs 48–72 h to enter in ketogenic state following a PSMF with the consequent appetite suppression [34, 35].

### Pulmonary function test

In the present experience, a clear benefit for lung function has been demonstrated. As a matter of fact, the respiratory function test FRC and ERV (espiratory reserve volume significantly improved after only 10 days of PSMF by tube, demonstrating the efficacy of this regime in improving the respiratory muscle contraction in the short period. In the literature, the most striking effect of weight loss in morbid obesity has been the increase in lung volumes, particularly in ERV or FRC. Several studies have reported a significant increase in maximal voluntary ventilation (MVV). Anyway the changes in FRC reflect changes in ERV and the improvement in oxygenation is assumed to result from the increase in ERV.

Various authors report important % increase of ERV from the baseline, but these data are significant particularly following important weight reduction. (ERV +157 % from the baseline after BMI decrease from 49 to 33,7; ERV: +65 % after BMI decrease from 42.1 to 30.9 and ERV +48 % after BMI decrease from 56.7 to 37.4) [36–38].

Functional residual capacity is the volume of air in the lungs at the end of a normal expiration when all respiratory muscles are relaxed. It physiologically represents the most important lung volume, because it approximates the level of normal breathing. The forces of elastic movement of the chest wall, directed outwards, tend to increase the lung volume, but are balanced by the forces of elastic movement of the lungs, directed inwards, which tend to reduce it. These forces are normally equal and opposite to a lung volume equal to about 40 % of TLC. The fast improvement of the FRC demonstrate a good response to PSMF on restrictive lung process due to obesity and is mainly due to the improvement of the ERV because it represents the volume of air remaining in the lungs at the end of a maximal exhalation (ERV = FRC − RV). Typically, the anomalous characteristic of obesity is a reduced ERV, due to a marked reduction of FRC with a relatively well preserved RV. 

The effect of rapid weight loss on pulmonary gas exchange has been evaluated mostly in post-bariatric surgery, in particular, according to Zavorsky and Hoffman [39] over 14 studies based on 288 subjects (age 37 years, BMI 45 kg m^2^).

Post-surgical data were measured up to 18.1 months (mean range = 2.5–47 months). Mean pre- and post-weight was 145 and 101 kg, respectively. The mean weight loss was 45 kg (mean range was from −21 to −62 kg) with a loss in BMI by 13 kg m^2^ (mean range = −7 to −24 kg m^2^) at 18.1 months post-operation, improvement in respiratory status was documented, with an increase in PaO_2_ by 10 mmHg (mean range = 1–23 mmHg), a reduction in AaDO2 by 8 mmHg (mean range = −3 to −16 mmHg) and PaCO_2_ by −3 mmHg (mean range = 3 to −14 mmHg) [39].

 In previous experiences weight loss improved lung function and oxygenation in obesity.

According to Hakala a marked rise in FRC and ERV with weight loss from 0.47 to 1.15 (*p* < 0.01) is observed after 6 weeks of very-low-calorie diet (VLCD) and behavioral intervention in morbid obesity with VLCD [Dietta Mini@ (MediFood, Finland)] composed of 2,100 kJ/day (500 kcal/day) (35 % protein, 1.8 % fat, 47 % carbohydrate) with 100 % of the recommended daily allowance of vitamins and minerals [40, 41]. A statistically significant improvement of lung function (PEF, FEV1, and FVC) has been observed also in obese asthma patients, before and after a VLCD period of 8 weeks [42]. 

The most significant improvement in lung volumes was the increase in ERV from 0.43 to 0.72 L. The rise in ERV also mainly accounts for the increase in FRC, because RV did not change [43].

Aaron et al. [44] after an average loss of 20 kg over a 6-month period, observed that, for every 10 % relative loss of weight, the FVC improved by 92 mL (*p* < 0.05) and the FEV1 improved by 73 mL (*p* < 0.04). Patients who lost >13 % of their pretreatment weight experienced improvements in FEV1 (*p* < 0.01), FVC (*p* < 0.02), and total lung capacity (*p* < 0.05) compared to patients in the lowest quartile who failed to lose significant amounts of weight. In the present experience, the PSMF without carbohydrates and lipids provokes a significant improvement of the PFT in a very short period after 10 days of weight reduction demonstrating how far this could influence lung function also when exogenous energy is completely reduced with comparable results to that of a post-bariatric surgery treatment. If we consider that there is a significant association between handgrip strength and FVC suggesting a further association between FVC and FFM, given that muscle tissue mass is counted in the FFM compartment, the maintenance of hand grip strength in our studied population suggest that the Respiratory muscle performance (muscle strength and endurance), could be improved after ProMoFastT.

### Enteral feeding or PSMF complications and side effects

There are no reported major complications and side effects resulting from the enteral nutrition in any patient. In particular, no cardiac arrhythmias have been reported, nor diarrhea nor cramps.

Constipation (3/22; 13.6 %) instead of fiber treatment, dizziness or headache during the first 2 days [20/22; 90.9 % (only two needed medication)], general weakness (3/22; 13,6 %), halitosis (5/22; 22,7 %) controlled by sugarless mint chewing-gum. The naso-gastric tube was blocked in 3 patients and required repositioning. 

Most of the people felt very well and full of energy compared with the period before.

As a matter of fact in the past, with PSMF have been described ventricular arrhythmias, which might lead to death [45, 46]. A correct analysis of these old data demonstrated that almost all of those patients were using hydrolyzed collagen as a protein source and were not taking vitamin and mineral supplements [45]. Other investigators reported arrhythmias in a prospective study of patients using hydrolyzed collagen without adequate vitamin and mineral intakes [46].

Hydrolyzed collagen is very poor quality protein, and some investigators suspect that there could be cardiotoxins in this hydrolysate of leftover, nonedible animal parts (tendons, ligaments, hide, etc.). In particular, recent studies, using PSMF with high-quality protein and RDA levels of vitamins and minerals have not shown arrhythmias on repeated 24-h electrocardiographic (Holter) monitoring [45, 47]. In the present experience, high-quality casein was used (resource instant protein Nestlé Health Science).

All the patients had a slight physiologic ketosis during the 10 days ProMoFasT. 

As far as ketosis is concerned, it is important to underline the difference between diabetes and fasting. The two conditions are opposite: in diabetes “fuel” is present, i.e., glucose and what is missing is the “access key” to the cells or rather insulin; while in fasting the situation is the opposite. 

Therefore, even though during fasting the counter-regulatory hormones are more active, insulin is still present.

## Conclusions

The analysis of the obtained data shows that the method of enteral feeding with protein-sparing modified fast ready in enteral formulation by tube) (ProMoFasT)over the 24 h has got no side effects and it is well tolerated by patients; therefore, it is safe and acceptable. After 10 days of treatment there is a significant weight loss which is maintained for 30 days, after 20 days of a balanced mildly reduced calorie diet. Body composition measured through bioimpedance methodical, reveal a significant decrease of fat free mass after 10 days which can be interpreted by a hydration increase immediately followed by a rapid increase in fat free mass after 20 days of hypocaloric well-balanced diet. Regarding the analysis of the VAS appetite, there is a significant reduction at 10 days, with a more marked reduction after 30 days. With regard to pulmonary function tests, contrary to what was expected, there is not only a failure to reduced lung function or a simple maintenance, but also a substantial improvement shown by the significant difference in FRC before and after 10 days and the ERV (respiratory reserve volume) in both groups suggesting a significant improvement of respiratory function demonstrated for the first time after 10 days of PSMF with or without tube.

The trend to improvement of hand grip strength observed, which is positively correlated, in literature, to respiratory muscle performance (muscle strength and endurance), suggests that the PSMF with naso-gastric tube could determine a fast respiratory benefit in the obese patient. The absence of complications and side effects and the maintenance of fat free mass let us make some considerations about the correct protocol which must be followed. From the nutritional point of view, it is mandatory to administer an amount of proteins equal to 1.2 g/kg/day with adequate vitamin (RDA), electrolyte (RDA) and fibers (20 g/day) support. In conclusion, from the clinical point of view the ProMoFasT method appears safe and associated with significant weight loss related to decrease of fat mass and appetite.

 Therefore, this method could be considered a valuable alternative to other methods, such as the intragastric balloon, which have much more complications and side effects. The current pilot study suggests on one hand to implement the size of the sample by a randomized multicenter trial, and, on the other hand, to develop additional studies that analyze the enterohormonal response during treatment with tube and infusion on 24 h. In the light of the obtained results, possible clinical research field for the method could be the pre-operative bariatric, gynecological orthopedics and lymphatic surgery. Finally, it is crucial to note that proponents of treatment with ProMoFasT must possess appropriate skills in terms of:

- Experience in the diagnostic and therapeutic management of obesity;
- Experience in artificial nutrition;
- Organization of the home enteral nutrition with a standardized method with clear paths and management of potential complications;
- Taking care of the subjects in a multidisciplinary organization and therapeutic rehabilitation.

It is well known that the treatment of obesity needs long-term management consisting of a multidisciplinary therapeutic path in which the changes in lifestyle, throughout diet, physical activity and behavioral treatment could lead to concrete results which could be stable especially in maintenance.
